# Association of retinal nerve fiber abnormalities with serum CNTF and cognitive functions in schizophrenia patients

**DOI:** 10.7717/peerj.9279

**Published:** 2020-06-02

**Authors:** Yanhong Liu, Lvzhen Huang, Yongsheng Tong, Jingxu Chen, Dongfang Gao, Fude Yang

**Affiliations:** 1 Peking University Huilongguan Clinical Medical School, Beijing Huilongguan Hospital, Peking University, Beijing, China; 2 People’s Hospital of Peking University, Peking University, Beijing, China

**Keywords:** Schizophrenia, Retinal nerve fiver, CNTF

## Abstract

**Background:**

Recent studies have reported reductions in retinal nerve fiber layers (RNFL) in schizophrenia. Ciliary neurotrophic factor (CNTF) has shown protective effects on both the neurogenesis and retina. This study aimed at investigating retinal abnormalities and establishing their correlation with serum CNTF and cognitive impairments in schizophrenic Chinese patients.

**Methods:**

In total, 221 patients diagnosed with schizophrenia and 149 healthy controls were enrolled. Serum CNTF and clinical features of patients were investigated. Cognitive functions were evaluated with Repeatable Battery for the Assessment of Neuropsychology Status (RBANS). RNFL thickness and macular thickness (MT) of both eyes were measured with optical coherence tomography (OCT). T-tests and analysis of covariance were used to compare the variables between the patient and control groups, while multiple linear regression analysis was performed to determine the associations of RNFL thickness, CNTF and cognitive impairments.

**Results:**

RNFL was found thinner in patients than in healthy controls (right: 88.18 ± 25.84 µm vs.102.13 ± 14.32 µm, *p* = 0.001; left: 92.84 ± 13.54 µm vs.103.71 ± 11.94 µm, *p* < 0.001). CNTF was lower in the schizophrenia group (1755.45 ± 375.73 pg/ml vs. 1909.99 ± 368.08 pg/ml, *p* = 0.001). Decline in RNFL thickness was found correlated with course of illness and serum CNTF in patients (all *p* < 0.05). Similarly, cognitive functions such as immediate memory and visuospatial functions were also found correlated with decline in RNFL thickness.

**Conclusion:**

Decline in RNFL thickness was associated with cognitive impairments of schizophrenia and CNFT serum concentration. The possibility of reduction in RNFL thickness as a biomarker for schizophrenia needs to be further examined.

## Introduction

Schizophrenia has been recognized as an abnormal development of brain networks. Involvement of neurodevelopmental mechanisms in the etiology of schizophrenia has been supported by various magnetic resonance imaging (MRI) and electrophysiological studies (Lavoie, Maziade & Hébert, 2014; Di et al., 2019; Zhang et al., 2019). The apoptosis or blocked development of neurons can be manifested as thinning of gray matter, enlargement of ventricles and the deepening of sulci in childhood-to-adolescence-onset schizophrenia (Gao et al., 2019).

Retina emerges from the diencephalon and gradually differentiates into the neural components of the eyes (Chu et al., 2012). Eyes transmit images of the outside world to the brain via parallel processing (Wässle, 2004). Effects of structural and functional changes in the brains of schizophrenia patients can also reflect in the retina. Therefore, the retina, as part of the whole nervous system and an easily accessible substrate for investigating brain pathology (Adams & Nasrallah, 2018), may potentially be associated with the complex etiology of schizophrenia (Schönfeldt-Lecuona et al., 2020). Observation of the retinal nerve structure and exploration of its correlation with cognitive impairments may contribute to a better understanding of the pathogenesis of schizophrenia.

Optical coherence tomography (OCT) is a fast and noninvasive imaging technology for displaying the retinal structure with an axial resolution of 5 µm or less (Hu, You & Zhang, 2015). Previous research using OCT has shown abnormal retinal structure (Silverstein & Rosen, 2015), including atrophy of the macula and the retina nerve fiber layers (RNFL), in patients with Alzheimer’s Disease (AD) (Moschos et al., 2012) and other neurological diseases related to cognitive impairments such as Parkinson’s disease and multiple sclerosis (Satue et al., 2014; Behbehani et al., 2015).

Cognitive impairments in schizophrenia, such as damage to working memory, have been related to the dysfunction of hippocampus (Grimm et al., 2018; Bygrave et al., 2019). On the other hand, previous studies have identified the promoting effect of ciliary neurotrophic factor (CNTF) on the repair of hippocampal neurons (Semkova, Häberlein & Krieglstein, 1999; Liu et al., 2003). Since serum nerve growth factor has protective effects on regional gray matter atrophy in schizophrenia (Neugebauer et al., 2019), thus it is reasonable to postulate that CNTF is one the factors for neuron nutrition that may in turn prevent impairments in RNFL and cognition.

However, associations among CNTF, RNFL and cognitive impairments in schizophrenia are not yet clear. Furthermore, association of RNFL abnormalities with the progression of schizophrenia also needs to be investigated scientifically. On the basis of neurodevelopmental hypothesis, CNTF can be a potential protective factor of schizophrenia (Lavedan et al., 2008); however, it needs to be further investigated. This study was designed to investigate retinal structural abnormalities and their correlations with CNTF and cognitive function in Chinese inpatients with schizophrenia. It was hypothesized that schizophrenia patients may show reduced RNFL thickness and that attenuated thickness may possibly be linked to lower levels of CNTF and cognitive impairments in schizophrenia.

## Materials and Methods

### Recruitment of subjects

Inpatients with schizophrenia and normal controls in 18–65 years age range were consecutively enrolled in Beijing Huilongguan Hospital from July 2018 to May 2019. Written informed consent was provided by participants or their guardians. Schizophrenia was diagnosed in patients according to the Clinical Interview for Diagnostic and Statistical Manual of Mental Disorders, 4th version (DSM-IV, American Psychiatric Association, APA). Normal controls were voluntarily recruited from hospital staff and nearby communities. Normal controls had no current or previous psychiatric history according to the structured clinical interview for DSM-IV (SCID-I). Proper informed consents were obtained all subjects or their guardians. The study was approved by Institutional Review Board of Beijing Huilongguan Hospital (number 2018-33).

### Exclusion criteria

Subjects with acute and chronic medical conditions, including inflammation, pregnancy, hypertension, head trauma, diabetes mellitus, drug and alcohol abuse or addiction, glaucoma, cataracts, high astigmatism (sphere diopter >4 and cylinder diopter >2), and optic media opacities were excluded from both the groups.

### Screening for current clinical features

Subjects in both groups were screened for their mean arterial pressure (MAP) and body mass index (BMI). Similarly, current three months smoking habits of all the subjects were recorded in order to consider smoking habit effect on retina. Positive family history of schizophrenia was recorded for the subject in patients’ group. Daily doses of antipsychotics taken by each patient were collected according to the medical records and were then converted to the olanzapine equivalent dose (OED) (Jonas et al., 2014; Meier et al., 2015).

### Measurement of serum CNTF

Blood samples from both the groups were collected at 6.30 a.m. after an overnight fast. Blood was allowed to coagulate at room temperature for 10–20 min followed by centrifugation at 300 RPM for 10 min. Serum was separated immediately stored at −80 °C till further analysis. Serum CNTF was measured with 96-well ELISA kits (Abcam, USA) following the manufacturer’s instructions (Nunes et al., 2018). To prevent the effects of metabolism on the optic nerve and vessels, levels of relevant variables such serum glucose, cholesterol, uric acid and alanine aminotransferase were measured and statistically controlled (Shiba et al., 2017). All the tests were performed in duplicate by certified technician blinded to the study.

### Measurement of cognitive functions

Cognitive impairments were measured in terms of Attention, Language, Visuospatial/Constructional, Immediate Memory, Delayed Memory and Total Scale using Repeatable Battery for the Assessment of Neuropsychology Status (RBANS). RBANS is a brief battery assessment for cognitive functioning originally used in dementia (Randolph et al., 1998). It comprises 12 subscales that contribute to six indices: Attention, Language, Visuospatial/Constructional, Immediate Memory, Delayed Memory and Total Scale. It is now commonly used in the assessment of cognitive impairment of schizophrenia (Man et al., 2018; Wang et al., 2019).

### Ophthalmic examination and measurement of RNFL and MT

All patients and normal controls were subjected to comprehensive ophthalmic examination by ophthalmologists. Best corrected visual acuity of both the eyes was measured through Snellen visual acuity. Anterior slit lamp microscopy, Goldman tonometer, and 90D focusing lens were used to exclude eye diseases and measure intraocular pressure. All the subjects underwent scan for measuring RNFL and Macular thickness (MT). For this purpose, Spectral domain OCT (Topcon 2002 type, Japan) scan was performed by ophthalmologist for both eyes of subjects. Clear images of the retina were captured with autofocused OCT camera (axial resolution5 µm; lateral resolution 10 µm). For RNFL, 3D data were obtained with an OCT raster centered on the optic nerve head in the “6 mm × 6 mm” cube with a 512 × 218 scan and 200 pixels. OCT software (version 8.42) automatically focused on the center of the disc and then extracted a circle with a diameter of 3-4 mm around the papilla to measure the RNFL thickness. RNFL thickness was measured at the 12 clock positions and in the four 90° quadrants of the whole eyeball, including the superior, inferior, nasal and temporal quadrants. The macular region was scanned in a radial “6 mm × 6 mm” region at 1024 × 12 and 200 superpixels. According to the grid definition by the Early Treatment Diabetic Retinopathy Study (ETDRS), a central circle (the foveal region), an inner ring and an outer ring were each divided into four quadrants with the OCT centered automatically on the fovea (Ascaso et al., 2015). MT was measured from the inner limiting membrane (ILM) to the retinal pigment epithelium (RPE). Total and average MT and RNFL thickness (microns) were all automatically calculated. Additionally, the disc area and cup volume were directly output from OCT.

### Statistical analysis

SPSS 20.0 software was used for analysis of data. The Kolmogorov–Smirnov test was used to confirm whether the data followed a normal distribution. Comparisons of demographic variables between patient and control groups were performed with independent sample t-tests and the chi-square test. Analysis of covariance was used to compare the thickness of RNFL and MT between patients and controls, adjusted for age, sex, OED and smoking. Multiple linear regression analysis was used to assess the thickness of RNFL and MT with the effects of illness duration and CNTF in the patients after controlling for age, sex, MAP, OED, smoking habits, positive family history, uric acid, glucose, and cholesterol levels. RNFL or MT was analyzed as a dependent variable. Illness duration, CNTF and all above controlled variables were analyzed as independent variables. Then, backward stepwise analysis was performed. Multiple linear regression analysis was also used to assess the cognitive function with the effects CNTF and RNFL in the patients after controlling for age, sex, smoking and duration of illness. The indices of RBANS were analyzed as dependent variables, and CNTF and RNFL were analyzed as independent variables. A *p* < 0.05 was considered statistically significant.

## Results

A total of 221 patients with schizophrenia and 149 healthy controls were enrolled in this study. Demographic characteristics of the enrolled subjects are summarized in Table 1. There were significant differences in age (44.20 ± 12.02 vs.41.59 ± 9.37 y, *p* = 0.004), sex and smoking habits between patients and controls (*p* ≤ 0.001). Clinical features such as MAP and BMI of subjects both in schizophrenia patients and control groups did not show any significant difference as shown in Table 1. The levels of serum uric acid and cholesterol were significantly higher in schizophrenia patients group (*p* = 0.012, *p* < 0.001), while level of serum glucose was lower in schizophrenia patients as compared to subjects in control group (*p* = 0.015). Results further showed that serum CNTF level was significantly lower in schizophrenia patients as compared to subjects in control group (1755.45 ± 375.73vs. 1709.99 ± 368.08 pg/ml, *t* =  − 3.347, *p* = 0.001).

**Table 1:** Participant demographics and differences in cognitive function between schizophrenia and normal groups.

Mean ± SD	Schizophrenia group	Control group	*t*∕*χ*^2^	*p*
Age, y	44.20 ± 12.02	41.59 ± 9.37	2.964	0.004
Sex Female, *n*	98	54	24.49	0.001
Male, *n*	123	95		
Duration of illness, y	20.11 ± 12.45	–		
Smokers, *n* (%)	76(23.7)	29(9.4)	22.985	<0.001
BMI (kg/m^2^)	24.23 ± 4.40	23.94 ± 3.34	0.434	0.164
Positive family history, *n* (%)	76	0		
Eye pressure left (mmHg)	15.39 ± 3.32	15.39 ± 2.70	0.009	0.993
Right (mmHg)	15.39 ± 3.33	15.24 ± 2.51	0.567	0.571
Diopters(D) left eye	3.07 ± 1.75	2.93 ± 1.14	0.255	0.800
Right eye	3.14 ± 1.83	2.97 ± 1.32	0.265	0.793
Olanzapine equivalent dose (mg/day)	2.58 ± 0.65	–	–	–
SBP^a^ (mmHg)	111.75 ± 9.16	110.85 ± 8.46	1.234	0.218
DBP^b^ (mmHg)	71.28 ± 5.42	72.10 ± 5.98	−1.752	0.083
MAP^c^ (mmHg)	84.77 ± 6.05	85.02 ± 6.30	−0.486	0.627
ALT^d^ (U/L)	22.09 ± 20.28	20.16 ± 15.18	1.092	0.275
AST^e^ (U/L)	19.73 ± 8.46	19.31 ± 7.91	0.538	0.591
Glucose (mmol/L)	5.12 ± 1.16	5.43 ± 1.52	−2.451	0.015
Cholesterol (mmol/L)	4.78 ± 0.84	3.99 ± 0.89	−9.489	<0.001
Triglyceride (mmol/L)	1.58 ± 0.80	1.50 ± 1.25	0.829	0.408
UA^f^ (mmol/L)	344.66 ± 98.16	321.70 ± 84.03	2.515	0.012
CNTF (pg/ml)^g^	1755.45 ± 375.73	1909.99 ± 368.08	−3.347	0.001
RBANS^h^				
Immediate memory	91.22 ± 15.92	106.84 ± 10.51	−9.306	<0.001
Visuospatial/Constructional	94.78 ± 15.21	105.69 ± 14.91	−5.969	<0.001
Language Index	92.91 ± 11.68	106.82 ± 9.55	−10.597	<0.001
Attention Index	104.12 ± 11.33	120.04 ± 16.56	−9.034	<0.001
Delayed Memory	91.98 ± 14.28	111.14 ± 7.86	−13.24	<0.001
Total Scale	92.16 ± 15.03	117.64 ± 12.13	−15.322	<0.001

**Notes.**

a Systolic blood pressure.

b Diastolic blood pressure.

c Mean arterial pressure.

d Alanine aminotransferase.

e Aspartate aminotransferase.

f Uricacid.

g CNTF:Ciliary.

h Neurotrophic factor Repeatable Battery for the Assessment of Neuropsychology Status (RBANS).

Retinal abnormalities were measured in terms of changes in MT and RNFL thickness and were investigated for their correlation with schizophrenia. As shown in Table 2, the RNFL thickness of both eyes in schizophrenia patients was found decreased when compared with that of normal controls (*p* < 0.001), however, the disc area and cup volume of both eyes’ retinas were not significantly different between the schizophrenia patients and subjects in control group. Although the average MT was not significantly different between the two groups, the MTs in the inner and outer rings of both eyes were slightly thinner in the patients group as compared to normal controls (*p* < 0.05). Results also revealed significant reduction in macula thickness of schizophrenia patients at the central region of the right macula and temporal quadrant of the left macula (*p* < 0.05).

**Table 2:** Comparison of retinal nerve fiber layer (RNFL) thickness and macular thickness in schizophrenia and normal control groups.

**Mean ± SD**	**Schizophrenia group**	**Control group**	***t***	***P***	***F***	***P*′**
**Right RNFL**						
Total thickness (µm)	88.18 ± 25.84	102.13 ± 14.32	−7.329	<0.001	12.052	0.001
Superior thickness (µm)	105.59 ± 36.68	120.13 ± 14.32	−4.521	<0.001	11.177	0.001
Inferior thickness (µm)	112.54 ± 37.91	130.17 ± 22.37	−6.169	<0.001	10.369	0.001
**Disc area (mm^2^)**	2.14 ± 1.10	2.34 ± 0.67	−2.283	0.023	0.848	0.358
**Cup volume (mm^3^)**	0.13 ± 0.13	0.11 ± 0.13	1.793	0.071	0.007	0.934
Left RNFL						
Total thickness (µm)	92.84 ± 13.54	103.71 ± 11.94	−8.637	<0.001	33.130	<0.001
Superior thickness (µm)	114.80 ± 27.62	128.55 ± 22.51	−5.622	<0.001	20.475	<0.001
Inferior thickness (µm)	117.49 ± 25.58	133.88 ± 18.68	−7.596	<0.001	15.183	<0.001
**Disc area (mm^2^)**	2.09 ± 0.90	2.24 ± 0.57	−2.088	0.037	0.086	0.770
**Cup volume (mm^3^)**	0.14 ± 0.13	0.11 ± 0.13	2.533	0.012	1.815	0.179
Right macula (µm)						
Average thickness	253.12 ± 37.42	263.82 ± 41.93	−3.162	0.002	0.052	0.820
Central thickness	217.65 ± 37.44	227.03 ± 23.87	−3.508	<0.001	8.173	0.005
Inner superior	277.85 ± 50.62	302.54 ± 16.22	−7.815	<0.001	19.199	<0.001
Inner inferior	272.27 ± 49.74	293.47 ± 19.67	−6.652	<0.001	9.396	0.002
Inner nasal	283.03 ± 44.02	298.85 ± 30.97	−4.902	<0.001	5.412	0.021
Inner temporal	269.21 ± 53.20	287.27 ± 15.86	−5.476	<0.001	2.570	0.110
Outer ring superior	251.01 ± 44.88	267.60 ± 14.81	−5.906	<0.001	9.336	0.002
Outer inferior	238.61 ± 43.52	255.79 ± 15.11	−6.271	<0.001	12.545	<0.001
Outer nasal	262.50 ± 49.28	283.07 ± 16.44	−6.660	<0.001	8.138	0.005
Outer temporal	232.36 ± 45.22	248.99 ± 16.62	−5.801	<0.001	3.628	0.058
**Left macula (µm)**						
Average thickness	254.67 ± 37.12	263.91 ± 36.37	−2.948	0.003	0.283	0.595
Central thickness	218.04 ± 36.98	224.19 ± 21.71	−2.386	0.017	2.860	0.092
Inner superior	281.47 ± 45.20	302.96 ± 17.16	−7.422	<0.001	17.543	<0.001
Inner inferior	273.53 ± 44.44	292.06 ± 20.18	−6.332	<0.001	16.942	<0.001
Inner nasal	284.27 ± 47.03	298.44 ± 26.10	−4.385	<0.001	6.100	0.014
Inner temporal	267.38 ± 40.36	282.70 ± 18.62	−5.749	<0.001	11.939	0.001
Outer superior	251.04 ± 40.32	267.11 ± 17.73	−6.088	<0.001	15.278	<0.001
Outer inferior	242.68 ± 38.20	255.43 ± 18.16	−5.025	<0.001	11.701	0.001
Outer nasal	264.94 ± 43.81	279.0 ± 25.18	−4.631	<0.001	7.566	0.006
Outer temporal	239.36 ± 36.31	247.96 ± 18.87	−3.501	0.001	4.442	0.036

**Notes.**

*P*, for *t*-test; *p*′, adjusted *p* for analysis of covariance after controlling for age, sex, olanzapine equivalent dose, and smoking.

Changes in MT and RNFL thickness were also investigated for their correlation with serum CNTF and duration of illness. Results revealed that MT was not significantly correlated with serum CNTF and duration of illness in patients with schizophrenia (*p* > 0.05). On other hand, changes in RNFL thickness revealed significant correlation with serum CNFT as well as duration of illness as shown in Table 3. Total thickness of RNFL of both eyes significantly decreased along with decline in serum CNTF and prolonged course of schizophrenia. To further authenticate the correlation of retinal abnormalities with schizophrenia, effects of changes in RNFL thickness on cognitive functions were investigated. The scores of immediate memory and visuospatial/constructional index apparently decreased with decline in RNFL thickness (*p*_1_ = 0.033; *p*_2_ = 0.008). Similarly, the score of language index slightly decreased with decline both in serum CNTF and RNFL thickness. Effects of serum CNFT on all other cognitive functions are shown in Table 4.

**Table 3:** Influence of CNTF and duration of illness on RNFL of schizophrenia patients (linear regression analysis).

	CNTF					Duration of illness				
	Constant	*β*	S.E.	*F*	*p*	Constant	*β*	S.E.	*F*	*p*
Left RNFL										
Total thickness	199.64	0.012	0.006	−2.220	0.032^a^	199.64	−0.657	0.192	−3.416	0.002^a^
Inferior thickness	100.68	−0.005	0.007	−0.773	0.442	111.02	0.040	0.339	0.118	0.907
Superior thickness	115.86	−0.633	0.378	−1.674	0.099	78.30	20.93	7.444	2.811	0.006
**Right RNFL**										
Total thickness	−47.63	0.018	0.008	2.327	0.023^b^	−47.63	−0.720	0.296	−2.433	0.017^b^
Inferior thickness	−73.31	0.015	0.011	1.353	0.180	−36.92	−1.059	0.434	−2.438	0.017^b^
Superior thickness	−73.97	−0.007	0.011	0.644	0.521	3.36	−1.127	0.412	−2.737	0.008^c^

**Notes.**

*Linear regression model using each RNFL and macula alone as a dependent variable and CNTF (ciliary neurotrophic factor) and duration of illness as independent variables after controlling for age, sex, MAP, OED, smoker, uric acid, positive family history, glucose, and cholesterol levels.

a MAP as a significant covariate in the equation.

b Sex and MAP as significant covariates in the equation.

c Sex and uric acid as significant covariates in the equation.

**Table 4:** Influence of CNTF and RNFL on the cognitive function of schizophrenia patients.

	CNTF					RNFL				
	Constant	*β*	S.E.	*F*	*p*	Constant	*β*	S.E.	*F*	*p*
Immediate memory	72.64	−0.001	0.007	−0.142	0.887	83.15	0.139	0.063	2.190	0.033
Visuospatial/Constructional	134.81	0.002	0.006	0.352	0.726	144.02	0.151	0.055	2.733	0.008^a^
Language Index	78.96	0.007	0.003	2.138	0.037	78.96	0.074	0.037	1.993	0.050
Attention Index	110.88	−0.003	0.005	−0.577	0.566	104.77	0.030	0.045	0.672	0.504
Delayed Memory	89.65	0.002	0.006	0.412	0.682	86.70	0.078	0.058	1.343	0.185
Total	102.06	0.001	0.007	0.090	0.929	103.25	0.035	0.070	0.501	0.619

**Notes.**

*Linear regression model using the Index of RBANS as a dependent variable and CNTF and right RNFL as independent variables after controlling for age, sex, duration of illness, and smoking.

a Age and duration of illness as significant covariates in the equation.

## Discussion

This study aimed at investigating the association of retinal abnormalities with serum CNTF level and other cognitive impairments in schizophrenia patients. Retinal abnormalities were measured in terms of changes in MT and RNFL thickness. Results of the current study revealed an uneven atrophy of MT and RNFL in schizophrenia patients. Moreover, a total thickness of RNFL in both the eyes of schizophrenia patients was found significantly correlated with decline in serum CNTF concentration. When further investigated in terms of the subarea of RNFL, only the inferior and superior RNFL thickness of the right eye was associated with the course of illness. Similarly, cognitive impairments were also found significantly related to reduced thickness of RNFL. Thus, reduction in RNFL thickness might be a possible biomarker of schizophrenia.

Studies have reported reduction in thickness of the RNFL in patients with schizophrenia (Pan et al., 2018; Samani et al., 2018; Silverstein et al., 2018). Atrophy of the macula and the RNFL was also reported among the patients with schizophrenia spectrum disorder (Schönfeldt-Lecuona et al., 2020). Findings of this study revealed decreased thickness of RNFL and macula in patients with schizophrenia which is in good agreement with results of the previously reported studies. Interestingly, reduction in RNFL thickness was found limited to the central macula of the right eye and the temporal quadrant macula of the left eye. Similarly, uneven reduction was also found in the two highly differentiated complex eyes in patients with schizophrenia spectrum disorder (Schönfeldt-Lecuona et al., 2020). The asymmetrical changes could be similar to the abnormal laterality (in terms of volume, connectivity or function alterations) found in the brains of patients with schizophrenia (Ribolsi et al., 2014). However, this is not in good agreement with another study reporting no significant atrophy in the RNFL, macula, or ganglion cell-inner plexiform layer thickness in schizophrenia patients (Silverstein et al., 2018), while the subgroup analyses showed significant atrophy in the “schizophrenia + comorbidity” group. The authors argued that the differences in structural retinal parameters reported in previous studies of OCT were possibly due to comorbidity with diabetes mellitus or hypertension. In case of our current study, abnormalities in the RNFL and the macula in patients with schizophrenia were clearly apparent even after controlling these confounding factors. Thus, it can be inferred that this reduction of RNFL may be the intrinsic characteristic change of schizophrenia patients. This in turn provides the base for considering reduction in RNFL thickness as possible cost-effective and non-invasive biomarker alone or in combination with other markers for early stage identification of schizophrenia in patients. Retinal structure abnormalities have been extensively reported as biomarkers for various other neurological conditions such as Parkinson’s disease, Alzheimer’s disease, amyotrophic lateral sclerosis, multiple sclerosis and prion diseases (Yap et al., 2019). However, further follow-up research should be carried out in high-risk relatives to clarify if the retinal changes in schizophrenia occurred as a genetic endophenotype.

Significant correlations were found between the course of illness and the thickness of the RNFL, but not MT. This result is supported by a previous study reporting a similar significant correlation between RNFL thickness and the duration of illness (Lee et al., 2013). The atrophy of the RNFL has been proved to indicate a predominant neuron loss in the retina (Asanad et al., 2019). This association between RNFL and the course of illness may indicate the change of RNFL occurs at a certain stage or all times of the course. The changes in RNFL during schizophrenia give new insights about retina as a component of nervous system for non-invasive and low-cost diagnosis of the diseases. Thus, early detection of this abnormality may possibly help the clinicians in the early identification and effective intervention and management of schizophrenia.

The reduction of the RNFL was significantly correlated with the impairments of immediate memory and visuospatial function in our study. The confounding effects of schizophrenia severity and medication on the reduction of RNFL remain unclear (Kazakos & Karageorgiou, 2020). After having excluded the antipsychotics interference, we still detected the thinning of the RNFL and its relationship with cognitive impairments. It has been already proved that cognitive impairments in dementia are also related to atrophy of the RNFL (Chan et al., 2019). The neurodegenerative hypothesis is proven in the etiology of AD. Nevertheless, the thickness of the RNFL in older healthy people is less correlated with cognitive function. In contrast, the relationship between higher levels of cognitive function and a thicker RNFL in young healthy people may reflect a better development process of tissues in the entire forebrain (Van Koolwijk et al., 2009). The cognitive impairments appear at the beginning of schizophrenia, including loss of working memory. Thus, it can be assumed that the decrease in the RNFL thickness is related to cognitive impairment in the onset period, and the thinning of the RNFL structure may be one of the neurobiological biomarkers. As the course of schizophrenia continues, the reduction may become more obvious. It is suggested that early intervention in the reduction of the RNFL may improve the cognitive impairments of patients with schizophrenia.

CNTF is considered one of the members of the interleukin-6 cytokine family and a neurotrophic cytokine that plays a nutritional and regulatory role in retinal cells (Flachsbarth et al., 2018). Our study showed that CNTF deficiency may cause a reduction in the retinal nerve in patients with schizophrenia. CNTF is a common extracellular factor that prevents excessive production of opsins, photoreceptors of outer segments, and 11-cis-retinal to protect rods and cones from photodamage (Li et al., 2018). Intraocular administration of CNTF has been found to attenuate photoreceptor degeneration and preserve retinal functions in animal research models of inherited or induced retinal disease (Ghasemi et al., 2018). Studies have suggested that the CNTF plays an important role in the regeneration and repair of the optic nerve after injury (Leibinger et al., 2009). The CNTF is known to be the only neurotrophic factor that can effectively promote the regeneration of retinal optic ganglia, while other neurotrophic factors have been thought to promote the regeneration of axons over long distances (Cui et al., 1999). Furthermore, the CNTF exhibited important protective effects against damage of hippocampal neurons (Liu et al., 2003). The alterations of the RNFL associated with decline in CNTF may represent one stage in the pathogenesis of schizophrenia, and the detailed changes and their relationship need further study. It will also be a new research direction in the future that intake of exogenous CNTF may retard the thinning of the RNFL and indirectly improve cognitive function in schizophrenia.

### Limitations

There several limitations associated with this study. First, this was a cross-sectional study; therefore, confirm conclusion for the associations of serum CNTF, course of illness and RNFL thickness cannot be made. Second, enrolled patients had an average chronic illness course of 20 years. Thus, it is not clear whether RNFL abnormalities occurred before the onset of schizophrenia. Third, all of the patients had received antipsychotic treatment; this may have confounded our results. Future studies in this regard should be carried out in patients with no past or current history of using antipsychotics. Fourth, the thickness of the macula obtained with Topcon SD-OCT2002 was not a single-layer ganglion cell thickness; rather it was a cumulative ganglion cell complex owing to the automatic measurement limitation (Kazakos & Karageorgiou, 2020). A specific non-manual model for macular stratification may be a better method for further study (Schönfeldt-Lecuona et al., 2020). With the improvement in spatial resolution of OCT devices, cohort studies can be more reliable for investigating pathological changes in the retinas of schizophrenia patients.

## Conclusions

By conducting a large-sample, single-center, cross-sectional study in chronic schizophrenia, our study suggests that RNFL thickness is associated with the course of illness in schizophrenia and the concentrations of serum CNTF. The reduction in RNFL thickness may be involved in the biological etiology of cognitive impairment among patients with schizophrenia and might also be a possible biomarker of schizophrenia. To authenticate retinal abnormalities as biomarkers for schizophrenia on mechanistic level, multi-centered studies employing multiple advanced techniques should be carried out in newly diagnosed schizophrenia patients.

##  Supplemental Information

10.7717/peerj.9279/supp-1 Data S1 Raw data

## Additional Information and Declarations

### Data Availability

The following information was supplied regarding data availability: Raw data is available as Supplemental File.
